# Single telomere length analysis in *Ustilago maydis*, a high-resolution tool for examining fungal telomere length distribution and C-strand 5’-end processing

**DOI:** 10.15698/mic2018.09.645

**Published:** 2018-08-07

**Authors:** Ganduri Swapna, Eun Y. Yu, Neal F. Lue

**Affiliations:** 1 Department of Microbiology & Immunology, W. R. Hearst Microbiology Research Center, Weill Cornell Medical College, New York, New York, United States of America.; 2 Sandra and Edward Meyer Cancer Center, Weill Cornell Medical College, New York, New York, United States of America.

**Keywords:** telomere, telomerase, STELA, Blm, Trt1, Ustilago maydis

## Abstract

Telomeres play important roles in genome stability and cell proliferation. Telomere lengths are heterogeneous and because just a few abnormal telomeres are sufficient to trigger significant cellular response, it is informative to have accurate assays that reveal not only average telomere lengths, but also the distribution of the longest and shortest telomeres in a given sample. Herein we report for the first time, the development of single telomere length analysis (STELA) - a PCR-based assay that amplifies multiple, individual telomeres - for *Ustilago maydis*, a basidiomycete fungus. Compared to the standard telomere Southern technique, STELA revealed a broader distribution of telomere size as well as the existence of relatively short telomeres in wild type cells. When applied to *blm*∆*,* a mutant thought to be defective in telomere replication, STELA revealed preferential loss of long telomeres, whose maintenance may thus be especially dependent upon efficient replication. In comparison to *blm*∆*,* the *trt1*∆ (telomerase null) mutant exhibited greater erosion of short telomeres, consistent with a special role for telomerase in re-lengthening extra-short telomeres. We also used STELA to characterize the 5’ ends of telomere C-strand, and found that in *U. maydis*, they terminate preferentially at selected nucleotide positions within the telomere repeat. Deleting *trt1* altered the 5’-end distributions, suggesting that telomerase may directly or indirectly modulate C-strand 5’ end formation. These findings illustrate the utility of STELA as well as the strengths of *U. maydis* as a model system for telomere research.

## INTRODUCTION

Eukaryotic chromosome ends, or telomeres, play critical functions in maintaining genome stability and controlling cell proliferation. Telomere DNA comprises numerous copies of a short repeat, which is G-rich on the 3’-end-containing strand (G-strand) and C-rich on the complementary, 5’-end-containing strand (C-strand). This telomere DNA nucleates the assembly of a special nucleoprotein structure at chromosome ends, which in turn allows the cells to distinguish the normal ends from abnormal double strand breaks (DSBs) and to avoid inappropriate telomere repair 1,2. Telomeres can display varying degrees of dysfunction. Some abnormal telomeres trigger a DNA damage response that induces growth arrest 3. Other, more fully “deprotected” telomeres, are fusogenic and can trigger cycles of genomic re-arrangement 4,5.

Notably, an adequate amount of telomere DNA is required to promote the formation of a functional telomere, and different degrees of telomere loss are associated with different severities of telomere dysfunction 3. However, despite its crucial importance, telomere DNA is difficult to maintain for two reasons. First, owing to the propensity of G-rich telomere DNA to adopt G-quadruplex (G4) or related structures, replication forks often stall in the telomere region, resulting in stochastic telomere truncation 6. Accordingly, efficient telomere replication depends critically on multiple helicases and recombinational repair proteins (e.g., BLM, WRN, RTEL, FEN1, RAD51, BRCA2 and DNA2) that help overcome replication barriers or stabilize stalled forks 6,7,8. Second, owing to the end replication problem, telomere DNA experiences progressive shortening following each round of replication 9. To compensate for this loss, most eukaryotes rely on telomerase, a cellular reverse transcriptase that extends the G-strand of telomeres 10,11, and primase-Pol α, a replicative polymerase that specializes in the synthesis of the C-strand 12, thereby converting the newly generated 3’-overhangs to mostly duplex DNA. Hence, telomere dynamics in a cell population are modulated by multiple telomere-lengthening and telomere-shortening mechanisms.

We are particularly interested in understanding the interplay between telomere replication and telomerase in sustaining telomere length. For this purpose, we have been exploring the Basidiomycete fungus *Ustilago maydis* as a useful model system 13,14,15. In comparison to the standard budding and fission yeast models, *U. maydis* has a number of advantages, especially in regard to the study of telomere replication. First, unlike budding and fission yeasts, *U. maydis* has a telomere repeat sequence (TTAGGG/CCCTAA) that is identical to the human sequence. This is attractive because the propensity of the telomere sequence to form G4-related structures is believed to underlie the special difficulty of replicating through telomere DNA. Given that different G-rich sequences may adopt different structures, the sharing of identical telomere sequence in *U. maydis* and mammals allows one to obviate a confounding variable. Second, we showed earlier that just like their mammalian counterparts, several *U. maydis *helicase and repair mutants exhibit apparent telomere replication defects. In particular, the *U. maydis* *rad51*∆*,* *brh2*∆ (~BRCA2 deletion), and *blm*∆ mutants all exhibit prominent telomere shortening even in telomerase-positive cells 13,14. In contrast, even though some budding yeast helicase and repair proteins have also been implicated in telomere maintenance, this role is typically more discernable in telomerase-negative cells - e.g., *Saccharomyces cerevisiae* *SGS1 *or* RAD51* deletion can accelerate senescence of telomerase-negative mutants, but does not cause telomere shortening in wild type cells 16,17. By characterizing the *U. maydis* helicase/repair mutants and *trt1*∆ (telomerase-null) separately and in combination, we showed that the helicase/repair genes probably promote telomere maintenance through overlapping pathways that are distinct from the telomerase pathway 15. For example, in contrast to the progressive shortening phenotype of *trt1*∆, telomeres are stably maintained in the helicase/repair mutants, and combining the helicase/repair mutations with *trt1*∆ triggers accelerated senescence and telomere loss.

**Figure 1:**
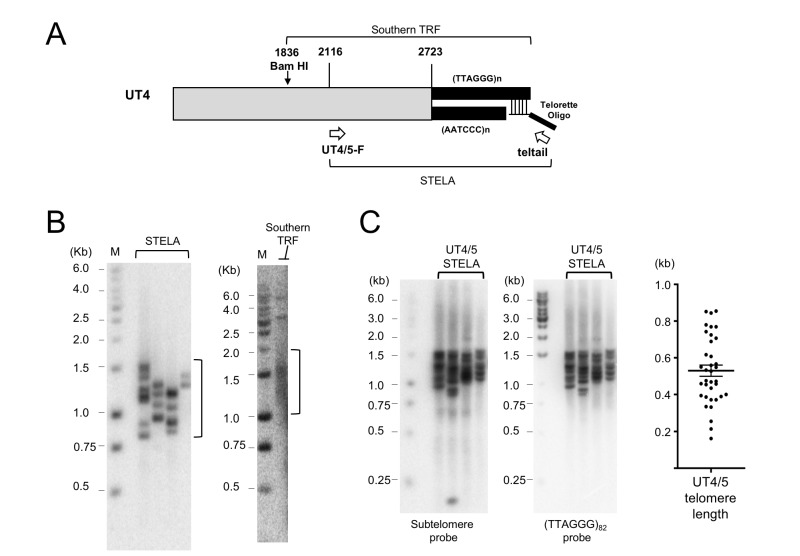
FIGURE 1: STELA protocol and investigation of UT4/5-containing telomeres. **(A)** Schematic illustration of the structure of UT4 and UT5-containing telomeres in *U. maydis*. The use of telorette oligos to modify the C-strand and the use of primers (UT4/5-F and teltail) to generate STELA products are also illustrated. **(B)** Four individual STELA PCR reactions for UT4/5 telomeres were performed using 2.5 pg of ligated wild type DNA as the template and shown on the left. A parallel Southern analysis is shown on the right. The same UT4/5 subtelomeric probe was used to detect telomere fragments in both analyses. **(C)** STELA assays were performed using 5 pg wild type DNA as the template, and the UT4/5-F and teltail oligos as primers. Following gel electrophoresis and transfer to a nylon membrane, the products were first detected using a UT4/5 subtelomeric probe (left panel). Subsequently, the UT4/5 probe was stripped from the membrane and the products re-analyzed using a TTAGGG repeat probe (middle panel). The sizes of the STELA fragments in the middle panel were determined using TESLA software. The lengths of the telomere tracts were then calculated by subtracting the subtelomere length (~630 bp), and then plotted (right). Error bars designate standard error of means.

In these previous examinations of telomere maintenance defects, we utilized Southern analysis of telomere restriction fragments (TRFs) as the assay for measuring telomere lengths. This long-standing, standard method suffers from several significant drawbacks owing to the size heterogeneity of the terminal repeat tracts, which vary by hundreds of base pairs. First, because the TRF clusters are visualized as smears in the assay, it is difficult to define accurately the upper and lower boundaries of these clusters. Second, because shorter telomere tracts yield weaker signals - as a result of binding fewer probes, the low range of the telomere distribution is prone to being underestimated. This is problematic because the telomere loss of the repair mutants may occur at only a small fraction of the telomeres, and because only a few abnormally short telomeres are sufficient to trigger a cellular response 3. To overcome the deficiencies of TRF Southern, investigators have sought to develop alternative methods for defining telomere length distributions 18. An example is single telomere length analysis (STELA), a PCR-based method initially developed to characterize human telomeres 19. In this assay, the 5’-end of telomere C-strand is first ligated to an anchor oligo, allowing the modified DNA to be subsequently amplified using a pair of primers that correspond to the anchor sequence and a chromosome-specific subtelomeric sequence, respectively (Fig. 1A). Variations of STELA were later developed to allow for simultaneous measurements of telomeres from all different chromosome ends 20,21. STELA has been particularly valuable in characterizing short telomeres, which, as noted before, are often under-identified. Interestingly, despite its potential utility, no STELA assay has been reported for either the budding or fission yeast telomeres. This is due in part to the irregular telomere repeats in both fungi, which render the design of anchor oligos challenging. While individual telomeres in *S. cerevisiae* have been investigated by cloning and sequencing 22, this labor-intensive strategy is not well suited to high throughput analysis of telomeres from many samples.

Because *U. maydis* has the same telomere repeat unit as mammals, the STELA protocol can be more readily applied to this fungus. Here we describe a modified STELA assay for *U. maydis* telomeres, which enabled us to detect individual chromosome ends bearing two types of subtelomeric repeats. Using this assay, we demonstrated a wider distribution of telomere sizes in wild type *U. maydis*, as well as the existence of relatively short telomeres. We also applied this assay to *blm*∆ and *trt1*∆ mutants, and found that the former exhibits a selective loss of long telomeres, whereas the latter manifests comparable erosion of both long and short telomeres. Moreover, we showed that the 5’ ends of *U. maydis* telomere C-strands preferentially terminate at several positions within the repeat, and these positions differ from that previously reported for mammals. Deleting *trt1* but not *blm* altered the distribution of telomere 5’ ends. These findings demonstrate the potential of the *U. maydis* STELA technique to provide new insights on telomere regulation.

## RESULTS

### STELA allows for measurements of individual telomere lengths in *U. maydis*

The STELA assay entails PCR amplification of individual telomeric fragments through the use of an anchor primer as well as a subtelomeric primer. Previous analysis of *U. maydis *subtelomeres revealed two classes of elements, named UTASa and UTASb. Individual members of UTASa include UT4 and UT5, whereas members of UTASb include UT6, UT7 and UT8 23. To generate STELA fragments from *U. maydis*, we first tested a subtelomeric primer that bears a sequence shared by the UT4 and UT5 subtelomeres, and that is located ~650 bp proximal to the TTAGGG terminal repeats (Fig. 1A) 24. Following PCR and Southern analysis using a UT4/5 probe, fragments that range in size from ~800 to 1,500 bp (average of 1,150 bp) were detected (Fig. 1B). Notably, in a parallel telomere restriction fragment (TRF) analysis, the same probe identified fragments with an average size of ~1.4 kb among *Bam*HI-digested genomic DNA (Fig 1B). Because a *Bam*HI site is located ~300 bp proximal to the UT4/5 forward primer, the average sizes of the STELA and TRF DNA are in good agreement with each other. Moreover, as expected, identical fragments were detected by the UT4/5 probe and the (TTAGGG)_82_ probe in a sequential hybridization experiment (Fig. 1C). Notably, the STELA reactions presented in Fig. 1C utilized higher amount of template DNA than reactions in Fig. 1B, leading to higher number of products and more similar size distributions for the different reactions. Also notably, relative to previous estimates 13, STELA revealed a slightly longer average telomere length (~500 bp) and greater telomere length heterogeneity (~150 to 850 bp, i.e., a maximal variation of 700 bp) for wild type* U. maydis *(Fig. 1C). These previous studies, unlike the TRF analysis in Fig. 1B, were based on probing PstI-generated TRF clusters using a telomere repeat (TTAGGG) probe, and estimated the average telomere lengths to be 300-400 bp or 400 bp 13,15. One of the studies also yielded a telomere size variation of ~ 300 bp 13. The discrepancies between these estimates from the current estimates are not surprising given the significant uncertainty involved in TRF-based telomere length analysis. In particular, it is difficult to judge the upper and lower boundaries of a TRF cluster (see e.g., the Southern analysis in Fig. 1B). The challenge becomes even more significant when there are multiple, overlapping TRF clusters, as was the case in the earlier studies 13,15.

**Figure 2:**
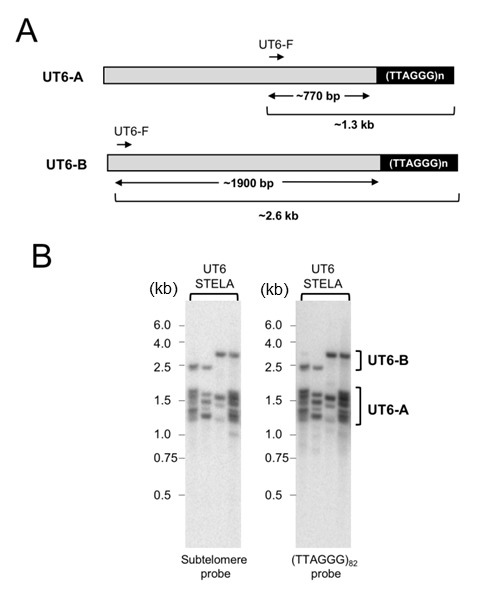
FIGURE 2: STELA analysis of UT6-containing telomeres. **(A)** Schematic illustration of two classes of UT6-containing telomeres in *U. maydis*. Note that the 770 bp estimate for UT6-A subtelomeres is based on our cloning and sequencing of STELA products and is considerably longer than the previously reported 519 bp estimate. **(B)** STELA assays were performed using wild type DNA as well as the UT6-F and teltail primers. Following gel electrophoresis and transfer to a nylon membrane, the products were first detected using a UT6 subtelomeric probe (left panel). Subsequently, the UT6 probe was stripped from the membrane and the products re-analyzed using a TTAGGG repeat probe (right panel).

To assess the generality of our initial findings, we utilized a different subtelomeric primer with a UT6-specific sequence (UT6-F) (Fig. 2A and 2B). Based on the literature, this primer is 519 bp proximal to the terminal repeats 23. However, as discussed below, cloning and sequencing of the STELA products suggests that the UT6 subtelomeres are longer (~770 bp) in our strain. Interestingly, probing the STELA products with either a UT6-probe or the (TTAGGG)_82_ probe revealed two clusters of products (designated as UT6-A and UT6-B in Fig. 2). Notably, the UT6-A products are ~ 5-fold more abundant than the UT6-B products. These observations suggest that in addition to the previously characterized UT6 element, there is a second, rarer type of UT6-containing telomeres in which the UT6-F primer is about 1.9 kb away from the terminal repeats.

To further confirm the accuracy of our deduction with regard to the sizes of the UT4 and UT6 subtelomere lengths in the STELA fragments, we cloned and sequenced a number of the PCR products (Supp. Fig. 1 and Supp. Fig. 2). Interestingly, we detected small sequence differences between all five UT4/5 and all five UT6 clones. For the UT4/5 clones, the sizes of the subtelomere segments range from 612 to 658 bp, close to that expected from a previous report (i.e., 612 bp 23). However, for the UT6 clones, the subtelomere segments are substantially longer than expected and range from 734 to 805 bp. While unexpected, this observation is consistent with our finding that the UT6-A STELA products are on average ~150 bp longer than the UT4 products (1.3 kb vs 1.15 kb). Notably, none of our UT6 clones harbor an ~1.9 kb subtelomere segment predicted for UT6-B, consistent with the lower abundance of this type of subtelomeric elements.

### The Blm helicase is critical for the maintenance of long, but not short telomere tracts

**Figure 3:**
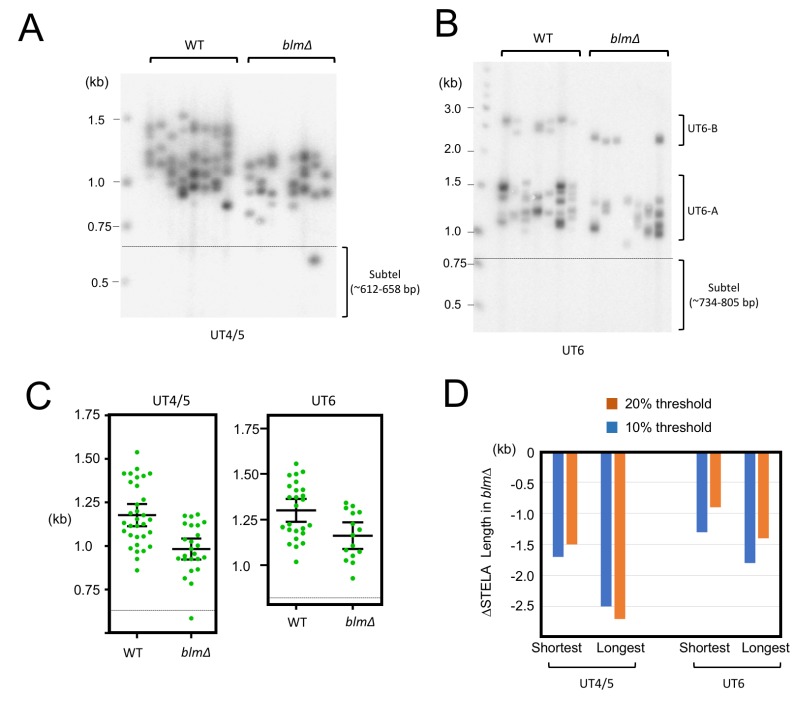
FIGURE 3: STELA comparison of telomeres in the wild type and *blm*∆ strains. **(A)** UT4/5 STELA assays were performed in parallel using wild type and *blm*∆ DNA. **(B)** UT6 STELA assays were performed in parallel using wild type and *blm*∆ DNA. **(C)** The STELA products in 3A and 3B were analyzed using the TESLA software, and the results plotted. Error bars designate standard errors of mean. **(D)** The telomere threshold lengths that mark the upper (or lower) quintile (20%) or decile (10%) of telomeres from the rest were determined for the various STELA samples. The reductions in the threshold lengths in *blm*∆ relative to wild type samples are then plotted.

The Blm helicase has been suggested to promote telomere replication by unwinding G-quadruplexes and related structures that form within the G-rich telomere repeats 25,26. We have previously observed significant telomere loss in the *U. maydis blm*∆ mutant, and estimated the average shortening to be ~220 bp 15. By subjecting *blm*∆ to STELA, we observed a striking defect of the mutant in the maintenance of long telomeres (Fig. 3). For example, in the UT4/5 assays, the *blm*∆ STELA samples did not contain any fragments in the 1.2-1.5 kb range. Such fragments, which contain ~550 to 850 bp of repeats, constitute close to half of the products in wild type samples (Fig. 3A and 3C). In contrast, the shortest UT4/5 STELA bands in both wild type and *blm*∆ samples are ~750-800 bp (corresponding to ~100-150 bp of telomeres). The complete absence of very long telomeres was also observed in the UT6 STELA assays for *blm*∆ (Fig. 3B and 3C).

To quantify the effects of* blm* deletion on the maintenance of long and short telomeres, we determined the threshold STELA lengths for the upper and lower 10% and 20% of the products, and then calculated the changes in these values in *blm*∆ relative to the parental control (Fig. 3D). Consistent with preferential loss of long telomeres, we found that the upper threshold lengths for *blm*∆ exhibited more erosion than the lower threshold lengths (e.g., ~250 bp vs ~150 bp for the UT4 products). Because current evidence points to a role for Blm in promoting telomere replication, our findings suggest that efficient replication is most important for the maintenance of long telomeres. Notably, this observation does not imply a special replication mechanism or a special Blm function for the most distal portion of the telomeres (see Discussion).

### Telomerase is needed to maintain minimal telomere lengths and plays a role in modulating telomere C-strand 5’ end formation

**Figure 4:**
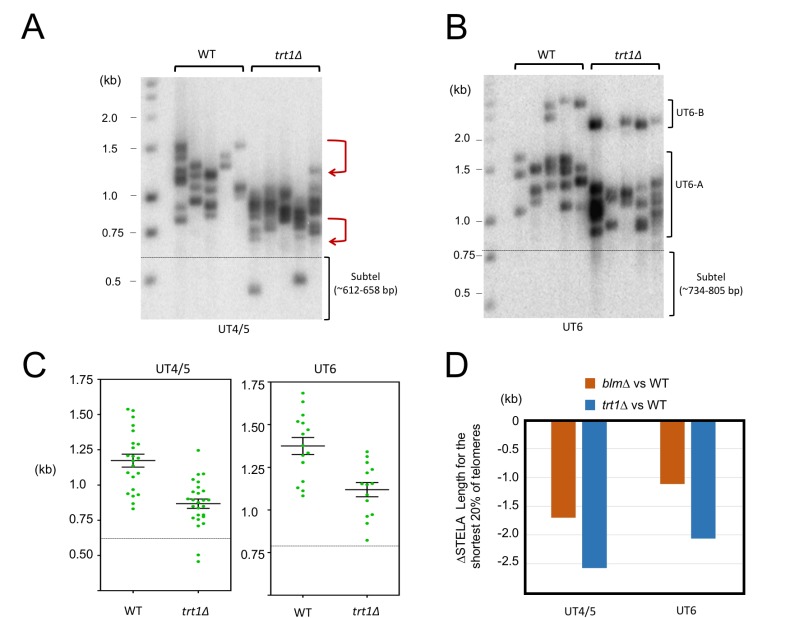
FIGURE 4: STELA comparison of telomeres in the wild type and *trt1*∆ strains. **(A)** UT4/5 STELA assays were performed in parallel using wild type and *trt1*∆ DNA. **(B)** UT6 STELA assays were performed in parallel using wild type and *trt1*∆ DNA. **(C)** The STELA products in 4A and 4B were analyzed using the TESLA software, and the results plotted. Error bars designate standard errors of mean. **(D)** The average lengths for the shortest 20% of the telomeres in the STELA analysis were determined for the wild type, *blm*∆, and *trt1*∆ samples, and the differences between the wild type and mutants plotted.

The preferential loss of long telomeres in *blm*∆ suggests the existence of compensatory mechanisms that allow for the maintenance of short but not long telomeres. Indeed, there is substantial evidence that telomerase preferentially elongates short telomeres 22. We therefore analyzed telomere distributions in the *trt1*∆ mutants by STELA (Fig. 4A, 4B and 4C). To minimize complications introduced by post senescent survivors, we isolated DNA from *trt1*∆ ~100 generations after gene deletion - senescence typically occurs at 200 generations. Compared to wild type telomeres, *trt1*∆ telomeres exhibit evident length reduction in both the upper and lower range of the size distribution (marked by two bent arrows in Fig. 4A). This is in contrast to *blm*∆*,* which manifests a more prominent reduction in the upper range. To confirm this visual impression, we calculated the average length for the shortest 20% of telomeres in the two mutants and compared them to that in the wild type strain (Fig. 4D). As predicted, the *trt1*∆ mutant suffered ~2-fold more telomere contraction than *blm*∆*, *supporting the notion that telomerase plays an important role in preventing the further erosion of short telomeres.

### *U. maydis* telomere C-strands terminate at preferred nucleotide positions within the 6-nt repeat unit

**Figure 5:**
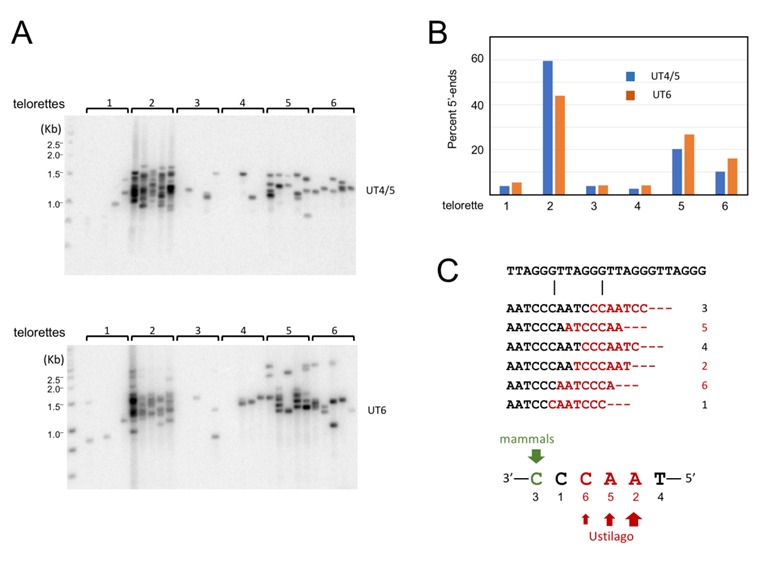
FIGURE 5: Characterization of the telomere C-strand 5’ end nucleotide in wild type *U. maydis*. **(A)** (Top) DNA from Wild type *U. maydis* was ligated separately to each of six individual telorette oligos, amplified using the UT4/5-F and teltail primers, and then subjected to Southern analysis. (Bottom) Wild type *U. maydis* DNA was ligated separately to each of six individual telorette oligos, amplified using the UT6-F and teltail primers, and then subjected to Southern analysis. **(B)** The number of UT4/5 and UT6 STELA products generated by each telorette oligos was determined and plotted as the percentage of total STELA products. **(C)** The position of the 5’ nucleotide identified by each individual telorette oligos is illustrated. Also indicated are the predominant *U. maydis* and mammalian 5’-end nucleotides.

In addition to revealing detailed telomere length distribution, STELA can be used to characterize the precise 5’-ends of the telomere C-strand because the ligation step requires precise juxtaposition of the 3’-end of telorette oligo and the 5’-end of C-strand 19,27. Through STELA analysis, mammalian telomere C-strands were previously shown to terminate at a preferred position with the telomere repeat, suggesting that the formation of the 5’ ends is subject to regulation 27,28. No comparable studies have been reported for any fungal telomeres. We therefore characterized the *U. maydis* telomere C-strand termini using individual telorette oligos (Fig. 5A). Interestingly, three of the six telorette oligos (2, 5, and 6) supported the amplification of substantially higher number of STELA products, suggesting that they define the preferred 5’-ends of C-strands. This observation applies to both UT4/5 and UT6 amplifications of ligated DNA (Fig. 5A). One of the three preferred ends, 5’-AACCCT (telorette 2), accounts for ~50-60% of the termini. The other two preferred ends, 5’-ACCCTA and 5’-CCCTAA, each accounts for ~10 to 20% of the termini (Fig. 5B). Notably, the three preferred 5’-ends are clustered together, suggesting that one underlying mechanism may be responsible. Also notably, these preferred 5’-ends for *U. maydis* C-strand are different from that defined for mammalian telomere, which is 5’-CTAACC 27 (Fig. 5C). Thus, while preferential termination at a specific position(s) is a conserved property of telomere C-strand, the identity of the preferred position is not conserved between fungi and mammals.

**Figure 6:**
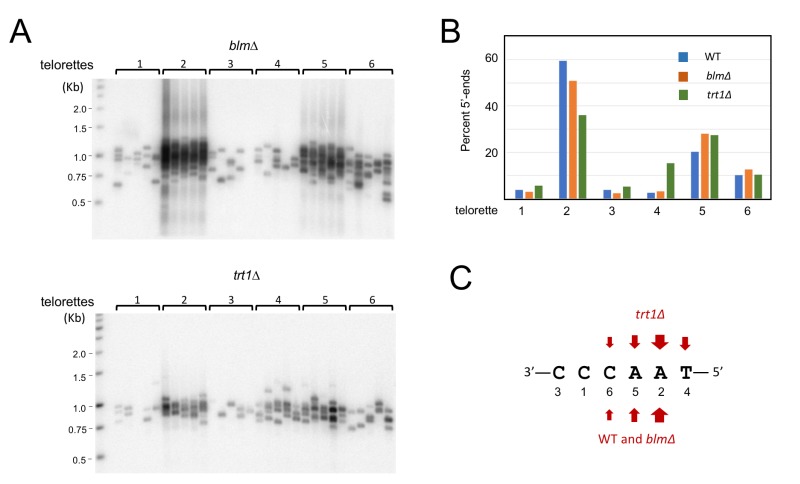
FIGURE 6: Characterization of the telomere C-strand 5’ end nucleotide in *blm*∆ and *trt1*∆. **(A)** (Top) DNA from *blm*∆ was ligated separately to each of six individual telorette oligo, amplified using the UT4/5-F and teltail primers, and then subjected to Southern analysis. (Bottom) DNA from *trt1*∆ was ligated separately to each of six individual telorette oligo, amplified using the UT4/5-F and teltail primers, and then subjected to Southern analysis. **(B)** The number of STELA products generated by each telorette oligo was determined for the *blm*∆ and *trt1*∆ DNA, and plotted as the percentage of total STELA products. **(C)** The predominant 5’ end nucleotides for the various *U. maydis *strains are indicated.

Next, we examined the impact of deleting *blm* or *trt1* on the distributions of C-strand 5’ ends on UT4/5 telomeres (Fig. 6). In comparison to the parental strain, only the *trt1*∆ mutant exhibited significant differences in its 5’ end distribution: the most predominant end in the parental strain (5’-AACCCT; telorette 2) became less well represented, and a rarely used end (5’-TAACCC; telorette 4) became more abundant (Fig. 6B and 6C). Most notably, the frequency of telorette 4 utilization is elevated from 2.5% in wild type DNA to 15.4% in *trt1*∆ DNA, representing a 6-fold increase. The observed alterations in the *trt1*∆ telomere 5’-ends were confirmed in three additional sets of STELA assays for the wild type and *trt1*∆ DNA; both the reduction in telorette 2 products and the increase in telorette 4 products in *trt1*∆ DNA were reproducible and statistically significant (Supp. Fig. 3). This result suggests that telomerase can directly or indirectly modulate C-strand 5’ end formation.

## DISCUSSION

In this study, we developed the first STELA assay for a fungus, and used it to characterize the telomere lengths and telomere 5’-end structures of this fungus. Our preliminary utilization of this assay to characterize the telomeres in the wild type and two mutant strains revealed a number of interesting findings as well as discrepancies from previous studies. The implications of these observations are discussed below.

### Comparison of the STELA results with previous characterizations of telomere lengths and subtelomeric structures

The size range of telomere repeat tracts as determined by STELA (a maximal variation of 700 bp) is significantly broader than that by Southern analysis (e.g., a maximal variation of about 300 bp in one study 15). This is not surprising given the limited resolution of the TRF analysis, and the uncertainty in marking the upper and lower boundaries of a TRF cluster. Moreover, because a short telomere yields weaker hybridization signal in Southern analysis, its presence is more likely to go undetected. In light of these considerations, it is notable that we found the minimal telomeres in the wild type *U. maydis* strain to be ~ 150 bps, suggesting that at this length, telomeres are recognized as sufficiently long to avoid obligatory extension by telomerase. STELA thus allows us to gain a better estimate of how telomerase activity is regulated by the length of telomeres.

One caveat of the STELA analysis is that it relies on PCR amplification to identify individual telomeres, and *a priori*, the PCR efficiency of individual telomeres may differ. However, two observations suggest that this is not a significant issue. First, in contrast to mammalian STELA fragments, which often vary by 5-10 kb in length in a given reaction, the *U. maydis *fragments are much more homogeneous in size. Second, most of the *U. maydis* STELA products in a given experiment have similar intensities, as would be predicted if they are amplified with similar efficiency. Importantly, there is no evidence that the shorter telomeres are more efficiently amplified; even the few STELA fragments that are evidently devoid of telomeric repeats (e.g., the *blm*∆ band below the dashed line in Fig. 3A) are amplified to the same level. Thus, the protocol we have developed appears to amplify the UT-4/5 and UT-6 telomeres in an unbiased manner, providing a fair representation of the total telomere population.

Previous analysis of *U. maydis *subtelomeres revealed two classes of elements, named UTASa (including UT4 and UT5) and UTASb (including UT6, UT7 and UT8). In our STELA analysis, we utilized two forward primers, one shared by UT4 and UT5, and the other specific to UT6 23. Cloning of the UT4/5 primer-derived products confirmed that these elements are highly similar to one another, and revealed additional variations in the length and sequences of these related elements. In particular, the sizes of the subtelomeric fragments as de-limited by the STELA forward primer and the TTAGGG repeats vary by ~ 50 bp. Such variations are also observed in the UT6 primer-derived products, which differ in length by as much as 70 bp. Moreover, we identified a rare variant subtelomere in which the UT6 primer is positioned about 1.9 kb away from the terminal repeat tract. Based on the relative abundance of STELA products, this rare variant is present at ~ 1/5 the frequency of the typical UT6 element. By further characterizing this and other rare variants, we may in the future be able to perform chromosome-specific STELA and characterize inter-chromosomal differences in telomere structure and dynamics, similar to what has been done for human telomeres 29.

### The distinct mechanisms of Blm and telomerase in promoting telomere maintenance 

A somewhat unique strength of *U. maydis* as a model system for telomere research is that like mammalian cells, mutations in *U. maydis* genes that promote telomere replication (e.g., *rad51*, *blm*) cause telomere loss in telomerase-positive cells 13,15. Such phenotypes have not been reported in comparable budding and fission yeast mutants. We have previously proposed that the telomere loss of these *U. maydis* mutants could be explained by stochastic truncation of telomeres due to fork stalling/collapse followed by incomplete re-extension by telomerase 15. The current analysis provides some additional insights on this balance. Most notably, the *blm*∆ mutant exhibits a dramatic and selective loss of long telomeres; telomeres with 550-850 bp repeat tracts, which are abundant in the parental strain, are completely missing in *blm*∆*.* This result suggests that complete replication of a telomere tract more than 550 bp long is problematic in the absence of Blm. In contrast, the minimal telomere length in the *blm*∆ mutant is similar to that of the parent strain. For example, in the UT4/5 STELA analysis, only 3 out 24 STELA fragments in the *blm*∆ sample have telomeres that are shorter than 150 bp (Fig. 3A and 3C), the minimal length found in the parental strain. The loss of very long telomeres in *blm*∆ and the retention of the 150 bp minimal telomere tracts do not necessarily imply a special Blm mechanism in the most distal portion of telomeres. First, it is possible that during replication, short telomere tracts are subjected to less frequent G4-related blockade, and hence less dependent on Blm for fork progression. Alternatively, telomerase may efficiently elongate very short telomeres and prevent their accumulation. Consistent with the second mechanism, when *trt1* is deleted, there is a substantial increase in the abundance of 50-150 bp telomeres, even in early passages (Fig. 4).

### Telomere C-strand maturation in *U. maydis*

A key advantage of the STELA assay is that it allows for the precise determination of telomere C-strand 5’ ends at individual chromosome termini. Previous examination of mammalian telomeres revealed the predominance of a specific 5’ end sequence (i.e., 5’-CTAACC), indicative of specific processing steps that favor the removal of more distal ribonucleotides and deoxyribonucleotides generated by primase-Pol (during C-strand synthesis 27,28. We performed a comparable analysis of *U. maydis* telomeres, and identified three preferred 5’ ends, which together account for ~80% of chromosome ends (Fig. 5). Interestingly, none of these ends correspond to the preferred mammalian telomere 5’ end. Thus, while preferential termination at a specific position(s) is a conserved property of telomere C-strand, the identity of the preferred position is not shared between fungi and mammals, even for fungi that bear the canonical telomere repeat sequence. This finding suggests that either the processing nucleases in these organisms have different properties, or that they are regulated differently by the telomere nucleoprotein structure.

It is notable that deleting *trt1* altered the distribution of the *U. maydis* C-strand 5’ end nucleotides. This is somewhat unexpected given that in the previous mammalian study, telomerase-positive and -negative cells were found to exhibit the same 5’-end preference 27. Since telomerase is not thought to be directly involved in C-strand formation, this effect in *U. maydis* may be mediated through an indirect mechanism. For example, it has been shown that in G-strand elongation, telomerase preferentially pause or terminate at the GGTTA**G** position, which is complementary to the last RNA template residue. This preferred G-strand terminus could indirectly influence the positioning of the last C-strand RNA-DNA chimera in the next cell cycle, or influence the positioning of G-strand-binding proteins such as Pot1. These positioning preferences could in turn modulate the eventual 5’ end of the C-strand. When telomerase is absent, the G-strand 3’ ends may become more heterogeneous, resulting in correspondingly greater heterogeneity in the C-strand 5’ ends. Additional studies will be required to test this and other interesting possibilities.

In summary, we have developed a STELA assay for *U. maydis*, and demonstrated the potential of this assay for characterizing telomere length distribution as well as the mechanisms of C-strand 5’ end formation. This represents the first STELA-based analysis for fungal telomeres, and provides further illustrations of the utility and versatility of *U. maydis* as a model system for investigating telomere mechanisms.

## MATERIALS AND METHODS

### *Ustilago maydis* strains and growth conditions 

Standard protocols were employed for the genetic manipulation of *U. maydis* 30,31,32. All *U. maydis* strains used in this study were haploid and were derived from the UCM350 background 31,33. These strains have all been described before and are listed in Supp. Table 1.

### Southern analysis of telomere restriction fragments

Southern analysis of telomere restriction fragments (TRF) was performed using DNA treated with *Bam*HI 13. The blot was hybridized to a labeled subtelomeric fragment generated by PCR using the UT4/5-F and UT4-subtel-R2375 primers (Supp. Table 2).

### STELA 

DNA from *U. maydis *strains was extracted using lysing enzyme and the GeneJET genomic DNA purification kit (Thermo Fisher Scientific). Ligation to telorette oligos (Supp. Table 2 and 27) were performed in 15 µl reactions containing 1 ng genomic DNA, 0.001 µM telorette oligo, 1x CutSmart Buffer (NEB), 1 mM ATP, and 800 U T4 DNA ligase (NEB) at 35°C for 20 hours. PCR assays were carried out in 25 µl reactions containing 2.5 - 10 pg template DNA, 1 µM each of the subtelomeric forward primer (UT4-F or UT6-F) and the teltail reverse primer, 1x Failsafe PCR PreMix H, and 2.5 U of Failsafe polymerase (Lucigen). Cycling conditions were as follows: 30 sec at 94°C, 30 sec at 63°C and 2 min at 72°C. 33 and 35 cycles were utilized for the UT4/5-telomere and UT6-telomere PCR reactions, respectively. To ensure adequate coverage of the telomere size distribution, we performed 4 - 8 parallel PCR reactions for each ligated DNA sample. The reaction products were analyzed by electrophoresis in 0.9 % agarose gels and subjected to Southern using the appropriate subtelomeric probes or a telomere repeat probe ((TTAGGG)_82_). Following PhosphorImager scanning (GE Healthcare), the sizes of individual telomeres were determined using TESLA software 20, and the results analyzed and plotted using Prism (GraphPad Software).

For cloning of STELA products containing UT4/5 and UT6 sequences, we increased the cycle numbers to 40 and 50, respectively. Following PCR amplification, the DNA was isolated using the Monarch® PCR & DNA Cleanup Kit (NEB, Inc.) and then introduced into the pMiniT 2.0 vector using the NEB® PCR Cloning Kit. The inserts were sequenced using the forward or reverse primer provided by the kit (pMiniT 2.0 forward and pMiniT 2.0 reverse, see Supp. Table 2).

## SUPPLEMENTAL MATERIAL

Click here for supplemental data file.

All supplemental data for this article are also available online at http://microbialcell.com/researcharticles/single-telomere-length-analysis-in-ustilago-maydis-a-high-resolution-tool-for-examining-fungal-telomere-length-distribution-and-c-strand-5-end-processing.
