# Supplementary material for: Single telomere length analysis in Ustilago maydis, a high-resolution tool for examining fungal telomere length distribution and C-strand 5’-end processing
Source: Microb Cell. 2018 Aug 7;5(9):393–403. doi: 10.15698/mic2018.09.645 (PMC6167521; doi:10.15698/mic2018.09.645)
Supplement: Supplementary file 1 [file mic-05-393-s01.pdf]

Supplemental Data

Ganduri et al.

Supp. Table 1. *U. maydis* strains used in this study

**Alias (Haploids)**	***Relevant Genotype***	**Reference**
UCM350[a]	wild type	Kojic et al., 2002
UEY12[ab]	*trt1*Δ	Yu et al., 2018
UCM693[ac]	*blm*Δ	Mao et al., 2009

[a] The genotype of UCM350 *is nar1-6 pan1-1 alb1*. *nar*, *pan*, and *ab* indicate inability to reduce nitrate, auxotrophic requirement for pantothenate, and mating type loci, respectively.
[b] *trt1* was disrupted by the insertion of *hph* cassette expressing the hygromycin resistance gene (Hyg^R).
[c] *blm* was disrupted by the insertion of *hph* cassette expressing the hygromycin resistance gene (Hyg^R).

Supp. Table 2. Oligonucleotides used in this study

**Oligo**	**Sequence 5' to 3'**
(For STELA)	
UT4/5-F	TCGGGCAACGTTCCATGTCG
UT4-subtel-R2375	CCCTCGAAGGCAGTGCATAC
UT6-F	CTACTACACATCGGTTCAGGC
UT6-subtel-R2400	ATGCCAAAGTGGAAATCGTGCAC
C Telorette 1	GCTCCGTGCATCTGGCATCCCCTAAC
C Telorette 2	GCTCCGTGCATCTGGCATCTAACCCT
C Telorette 3	GCTCCGTGCATCTGGCATCCCTAACC
C Telorette 4	GCTCCGTGCATCTGGCATCCTAACCC
C Telorette 5	GCTCCGTGCATCTGGCATCAACCCTA
C Telorette 6	GCTCCGTGCATCTGGCATCACCCTAA
Teltail	GCTCCGTGCATCTGGCATC
pMiniT 2.0 forward	ACCTGCCAACCAAAGCGAGAAC
pMiniT 2.0 reverse	TCAGGGTTATTGTCTCATGAGCG

```
                    UT4/5-F
UT4-a          1 TCGGGCAACGTTCCATGTCGAGTTCTCGGATGTGAACGAGTATATGCTCT
UT4-b          1 TCGGGCAACGTTCCATGTCGAGTTCTCGGATGTGAACGAGTATATGCTCT
UT4-c          1 TCGGGCAACGTTCCATGTCGAGTTCTCAGATGTGAACGAGTATATGCTCT
UT4-d          1 TCGGGCAACGTTCCATGTCGAGTTNTCGGATGTGAACGAGTATATGCTCT
UT4-e          1 TCGGGCAACGTTCCATGTCGAGTTCTCGGAGGTGAACGAGTATATGCTCT
consensus      1 ***********************.**.**.********************

UT4-a         51 GGGTGGGGACACCAGCAATGCTGGCAGGCAAGGAGTGCGTGATGGCCAAC
UT4-b         51 GGCTGGGGACACCAGCAATGCTGGCAGGCAAGGAGTGCGTGATGGCCAAC
UT4-c         51 GGGTGGGGACACCAGCAATGCTGGCAGGCAAGGAGTGCGTGATGGCCAGC
UT4-d         51 GGGTGGGGACACCAGCAATGCTGGCAGGCAAGGAGTGCGTGATGGCCAAC
UT4-e         51 GGGTGGGGACACCAGCAATGCTGGCAGGCAAGGAGTGCGTGATGGCCAAC
consensus     51 **.*********************************************.*

UT4-a        101 TGCGTTGCTGCAGCGCAACTGCAAGTGTGGGATCGACGGGATGATGACGA
UT4-b        101 TGCGTTGCTGCAGCGCAACTGCAAGTGTGGGATCGACGGGATGATGACGA
UT4-c        101 TGCGTTGCTGCAGCGCAACTGCAAGTGTGGGATCGACGGGATGATGACGA
UT4-d        101 TGCGTTGCTGCAGCGCAACTGCAAGTGTGGGATCGACGGGATGATGACGA
UT4-e        101 TGCGTTGCTGCAGCGCAACTGCAAGTGTGGGATCGACGGGATGATGACGA
consensus    101 **************************************************

UT4-a        151 AGAGCGGTGACAGGATCGCGAGCGGTGGTTGGTAAACACGGGGTTTGGAG
UT4-b        151 AGAGCGGTGACAGGAACGCGAGCGGTGGTTGGTAAACACGGGGTTTGGAG
UT4-c        151 AGAGCGGTGACAGGATCGCGAGCGGTGGTTGGTAAACACGGGGTTTGGAG
UT4-d        151 AGAGCGGTGACAGGATCGCGAGCGGTGGTTGGTAAACACGGGGTTTGGAG
UT4-e        151 AGAGCGGTGACAGGAACGCGAGCGGTGGTTGGTAAACACGGGGTTTGGAG
consensus    151 ***************.**********************************

UT4-a        201 CGGATCTGGATTTATTGCTGGACGCGGAGATTCGCTGCTCGTATGCGGTG
UT4-b        201 CGGATCTGGACGGGTTGCTGGACGCAGAGATCGGCTGTTCGTATGCACTG
UT4-c        201 CGGATCTGGATTTATTGCTGGACGCGGAGATTCGCTGCTCGTATGCGGTG
UT4-d        201 CGGATCTGGATTTATTGCTGGACGCGGAGATTCGCTGCTCGTATGCGGTG
UT4-e        201 CGGATCTGGATTTAATGCTGGACGCGGAGATTCGCTGCTCGTATGCGGTG
consensus    201 **********.....**********.*****..****.********..**

UT4-a        251 CCTTCGAGGGTGGATGCTGAGGGGTGGATGTATATTGTAGGTGGGTATGG
UT4-b        251 CCTTCGAGGGTGGATGCTGAGGGGTGGATGTATGTTGTAGGTGGGTATGG
UT4-c        251 CCTTCGAGGGTGGATGCTGAGGGGTGGATGTATATTGTAGGTGGGTATGG
UT4-d        251 CCTTCGAGGGTGGATGCTGAGGGGTGGATATATATTGTAGGTGGGTATGG
UT4-e        251 CCTTCGAGGGTGGATGCTGAGGGGTGGATGTATATTGTAGGTGGGTATGG
consensus    251 *****************************.***.****************

UT4-a        301 GGTGAAG----------TGGGGCAGATATATATTGTGAGTAAAACCATTA
UT4-b        301 GGTGAAG----------TGGGGCAGATATATATTGTGAGTAAAATCATTA
UT4-c        301 GGTGAAG----------TGGGGCAGATATATATTGTGAGTAAGATCATTA
UT4-d        301 GGTGAAG----------TGGGGCAGATATATATTGTGAGTAAAATCATTA
UT4-e        301 GGTGAAGATGGAGTAAGTGGGGCAGATATATATTGTGAGTAAAATAATTA
consensus    301 *******          *************************.*..****

UT4-a        341 TTTTTATTTGTATTTTAATTTTT-TT-TAATGTTGGTATTTAATTTGGAG
UT4-b        341 TTTTTATTTGTATTTTAATTTTTTTT-TAATGTTGGTATTTAATTTGGAG
UT4-c        341 TTTTTATTTGTACTTTAATTTTTTTT-TAATGTTGGTATTTAATTTGGAG
UT4-d        341 TTTTTATTTGTACTTTAATTTTTTTT-TAATGTTGGTATTTAATTTGGAG
UT4-e        351 TTTTTATTTGTATTTTAATTTTTTTTTTAATGTTGGTATTTAATTTGGAG
consensus    351 ************.**********.** ***********************

UT4-a        389 ATTATTAATTTTGTTTTGGTGGCAACATTGGGTGAGCGGTGAAGCGAGTC
UT4-b        390 ATTATTAGTTTGATTTTGGTGGCAACATTGGGTGAGCAGTGAAGGGAGTG
UT4-c        390 ATTATTAATTTTGTTTTGGTGGCAACATTGGGTGAGCGCTGAAGCGAGTC
UT4-d        390 ATTATTAATTTTGTTTTGGTGGCAACATTGGGTGAGCGCTGAAGCGAGTC
UT4-e        401 ATTAATAATTTTCTTTTGGTGGCAACATTGGGTGAGCACTGAAGGGAGTC
consensus    401 ****.**.***..************************..*****.****.

UT4-a        439 TGAGTGTGGCTGGGTATAGTGTGGGGGGTGAGTATGGTGGGTTGTTGTTG
UT4-b        440 TGAGTGTGGGTGGGTATAGTGTGGGGGGTGAGTATGGTGGGTTGTTGTTG
UT4-c        440 TGAGTGTGGCTGGGTATGGTGTGGGGGGTGAGTATGGTGGGTTGTTGTTG
UT4-d        440 TGAGTGTGGCTGGGTATGGTGTGGGGGGTGAGTATGGTGGGTTGTTGTTG
UT4-e        451 TGAGT----------------------------ATGGTGGGTTGTTCCTG
consensus    451 *****............................*************..**

UT4-a        489 GTGGCGGGCGGCAGCGCTTGAAGGGTTGGATGGAGGGGGGG-GTGATTGT
UT4-b        490 GTGGCGGGCTGCAGGGCTTGAAGAGTTGGATGGAG--GGGG-GTGATAGT
UT4-c        490 GTGGCGGGCGGCAGCGCTTGAAGGGTTGGATGGTGGGGGGT-GTGATTGT
UT4-d        490 GTGGCGGGCGGCAGCGCTTGAAGGGTTGGATGGAGGGGGGT-GTGATTGT
UT4-e        473 GTGGCGGGCTGCAGCACTTGAAGGGTTGGATGGAGGGGGGGGGTGATTGT
consensus    501 *********.****..*******.*********.*..***. *****.**

UT4-a        538 ATGTTGGTGTTGGGAGTGGGGATGGATGGGATGAGGAATTGAATGTGTCA
UT4-b        537 ATTTTGTTGTTGGGAGTGGGGATGGATGGGATGAGGGATTGAATGTGTCA
UT4-c        539 ATGTTGGTGTTGGGAGTAGGGATGGATGGGATGAGGGATTGAATTAA--G
UT4-d        539 ATGTTGGTGTTGGGAGCGGGGATGGATGGGATGAGGGATGGAATTAA--G
UT4-e        523 ATGTTGGTGTTGGGAGTGGGGATGGATGGGATGAGGGATTGAATGTGTCA
consensus    551 **.***.*********..******************.**.****......

UT4-a        588 ACGGGTGGAGCGCTGCAC------------------------CGCTGCAC
UT4-b        587 ACGGCTGGAGCGCTGCACCGCTGCACCGCTGCACCGCTGCACCGCTGCAC
UT4-c        587 ACGGCTGGAGCGCTGCAC--------------------------------
UT4-d        587 ACGGCTGGAGCCCTGCAC--------------------------------
UT4-e        573 ACGGGTGGAGCGCTGCAC--------------------------------
consensus    601 ****.******.******

UT4-a        614 GGCTGGACGGCTCAACCGCTGATAGGGTTA------
UT4-b        637 CGCTGGACGGCTCAACCGCTGATAGGGTTA------
UT4-c        605 GGCTGGACGGCTCAACCGCTGATAGGGATAGGGTTA
UT4-d        605 GGCTGGACGGCTCAACCGCTGATAGGGATAGGGTTA
UT4-e        591 GGCTGGACGGCTCAACCGCTGATAGGGTTA------
consensus    651 .************************.**...
                                       TTAGGG
```

Supp. Fig. 1. *Sequences of UT4/5 STELA products*
The sequences of 5 clones derived from a UT4/5 STELA reaction are aligned. Highlighted in yellow are the UT4/5-F primer and the start of the TTAGGG repeats in each clone.

UT6-F

```
UT6-a        1 ----------------------------------------CTACTACACA
UT6-b        1 ----------------------------------------CTACTACACA
UT6-c        1 CTACTACACATCGGTTCAGGCAGCGATCCNNCGC-C-ACGCTACTACACA
UT6-d        1 CTACTACACATCGGTTCAGGCAGCGATCCACCGCCCACA-CTACTACACA
UT6-e        1 CTACTACACATCGGTTCAGGCAGCGATCCCCCCNCCACTTTTATTACACA
consensus    1 .............................  .   .    .**.******

UT6-a       11 TCGGTTCAGGCATCGATCCTCACCGACCGACCGTCAACGCATGAGACCGA
UT6-b       11 TCGGTTCAGGCATCGATCCTCACCGACCGACCGTCAACGCATGAGTCCGA
UT6-c       49 TCGGTTCAGGCATCGATCCTCACCGACCGACCGTCAACGCATGAGACCGA
UT6-d       50 TCGGTTCAGGCATCGATCCTCACCGACCGACCGTCAACGCATGAGACCGA
UT6-e       51 TCGGTTCAGGCATCGATCCTCANCGACCGACCGTCAACGCATGAGACCGA
consensus   51 **********************.**********************.****

UT6-a       61 CACCGACACACTCTCTGACTGCAGCGGCTAAGCACAATGGCGTTCGACAC
UT6-b       61 CACCGACACACTCTCTGACTGCAGCGGCTAAGCACAATGGCGTTCGACAC
UT6-c       99 CACCGACACACTCTCTGACTGCAGCGGCTAAGCACAATGGCGTTCGACAC
UT6-d      100 CACCGACACACTCTCTGACTGCAGCGGCTAAGCACAATGGCGTTCGACAC
UT6-e      101 CACCGACACACTCTCTGACTGCAGCGGCTAAGCACAATGGCGTTCGACAC
consensus  101 **************************************************

UT6-a      111 CTTCCATCACAACACAGAGCATGGCGTTATTGTTTGTCGGCAGTGTGAGA
UT6-b      111 CTTCCATCACAACACAGAGCATGGCGTTATTGTTTGTCGGCAGTGTGAGA
UT6-c      149 CTTCCATCACAACACAGAGCATGGCGTTATTGTTTGTCGGCAGTGTGAGA
UT6-d      150 CTTCCATCACAACACAGAGCATGGCGTTATTGTTTGTCGGCAGTGTGAGA
UT6-e      151 CTTCCATCACAACACAGAGCATGGCGTTATTGTTTGTCGGCAGTGTGAGA
consensus  151 **************************************************

UT6-a      161 CCTGCCTGGGGTTAGGGTGCACGATTTCCACTTTGGCATCCACAT-CCAC
UT6-b      161 CCTGCCTGGGGTTAGGGTGCACGATTTCCACTTTGGCATCCACAT-CCAC
UT6-c      199 CCTGCCTGGGGTTAGGGTGCACGATTTCCACTTTGGCATCCACAT-CCAC
UT6-d      200 CCTGCCTGGGGTTAGGGTGCACGATTTCCACTTTGGCATCCACAT-CCAC
UT6-e      201 CCTGTCT-GGGTTAGGGTGCACGATTTCCACTTTGGCATCCACATTCCAC
consensus  201 ****.**.************************************* ****

UT6-a      210 CTCCACCCCAACGACGACTCGCACCTCCACACCAACGATGACCTGCACAT
UT6-b      210 CTCCACCCCAACGACGACTCGCATCTCCACACCAGCGATGACCTGCACAT
UT6-c      248 CTCCACCCCAACGACGACTCGCACCTCCACACCAACGATGACCTGCACAT
UT6-d      249 CTCCACCCCAACGACGACTCGCACCTCTACACCAACGATGACCTGCACAT
UT6-e      250 CTCCACCCCAACGACGACTCGCACCTCTACACCAACGATGACCTGCACAT
consensus  251 ***********************.***.******.***************

UT6-a      260 CCGCATCCACCTTGACGCTGAGCAGCTGGACGCCAGGGCGGAGCTGGACG
UT6-b      260 CCGCATCCACCTTGACGCTGAGCAGCTGGACGCCAGGGCGGAGCTGGACG
UT6-c      298 CCGCATCCACCTTGACGTTGAGCAGCTGGACGCCAGGGCGGAGCTGGACG
UT6-d      299 CCGCATCCACCTTGACGCTGAGCAGCTGGACGCCAGGGCGGAGCTGGACG
UT6-e      300 CCGCATCCACCTTGACGCTGAGCAGCTGGACGCCAGGGCGGAGCTGGACG
consensus  301 *****************.********************************

UT6-a      310 GGCAACTGAAGAAGGCGATCCGCGAGCTGTTGGTATCGGTCATGTCCTGT
UT6-b      310 AGCAACTGAAGAAGGCGATCCGCGAGCTGTTGGTATCGGTCATGTCCTGT
UT6-c      348 AGCAACTGAAGAAGGCGATCCGCGAGCTGTTGGTATCGGTCATGTCCTGT
UT6-d      349 AGCAACTGAAGAAGGCGATCCGCGAGCTGTTGGTATCGGTCATGTCCTGT
UT6-e      350 AGCAACTGAAGAAGGCGATCCGCGAGCTGTTGGTATCGGTCATGTCCTGT
consensus  351 .*************************************************

UT6-a      410 GGGCAGTGGATGATTGGTACAGCGGATGGACGGCAGATATATATTGTGAG
UT6-b      410 GGGCAGTGGATGATTGGTACAGCGGATGGACGGCAGATATATATTGTGAG
UT6-c      448 GGGCAGTGGATGATTGGTACAGCGGATGGACGGCAGATATATATTGTGAG
UT6-d      449 GGGCAGTGGATGATTGGTACAGCGGACGGACGGCAGATATATATTGTGAG
UT6-e      450 GGGCAGTGGATGATTGGTACAGCGGATGGACGGCAGATATATATTGTGAG
consensus  451 **************************.***********************

UT6-a      460 TGGAATATGATTTAAATTTTTAATTTTAATGT-TTTTTTTTTTAATTTTG
UT6-b      460 TGGAATATGATTTAAATTTTTAATTTTAATG-T-TTTTTTTTTAATTTTG
UT6-c      498 TGGAATATGATTTAAATTTTTAATTTTAATGTTTTTTTTTTTTAATTTTG
UT6-d      499 TGGAATATGATTTATATTTTTAATTTTAATGT-----TTTTTTAAGTTTG
UT6-e      500 TGGAATATGATTTATATTTTTAATTTTAATGT-----TTTTTTAATTTTG
consensus  501 **************.****************.  ...********.****

UT6-a      509 GTATTTAATTTGGAGATTATTAATTTGATTTTGGTGGCAACATTGGGTGA
UT6-b      508 GTATTTAATTTGGAGATTATTAATTTGATTTTGGTGGCAACATTGGGTGA
UT6-c      548 GTATTTAATTTGGAGATTATTAATTTGATTTTGGTGGCAACATTGGGTGA
UT6-d      544 GTAATTAATTTGGAGATTATTAGTTTGATTTTGGTGGCAACATTGGGTGA
UT6-e      545 GTAATTAATTTGGAGGTTATTAGTTTGATTTTGGTGGCAACATTGGGTGA
consensus  551 ***.***********.******.***************************

UT6-a      559 GCACTGAATGGAGT----------CTG------------------AGTAT
UT6-b      558 GCACTGAATGGAGT----------CTG------------------AGTAT
UT6-c      598 GCACTGAATGGAGT----------CTG------------------AGTAT
UT6-d      594 GCAGTGAAGGGAGTGTGAGTGTGGCTGGGTATAGTGTGGGGGGTGAGTAT
UT6-e      595 GCAGTGAAGGGAGTGTGAGTGTGGCTGGGTATAGTGTGGGGGGTGAGTAT
consensus  601 ***.****.*****          ***                  *****

UT6-a      581 GGTGGGTTGTTCTTGGTGGCGGGCTGCAGCGCTTGAAGGGTTGGATGGAC
UT6-b      580 GGTGGGTTGTTCTTGGTGGCGGGCTGCAGCGCTTGAAGGGTTGGATGGAC
UT6-c      620 GGTGGGTTGTTCTTGGTGGCGGGCTGCAGCGCTTGAAGGGTTGGATGGAC
UT6-d      644 GGTGGGTTGTTGTGGGTGGCGGGCTGCAGGGCTTGAAGAGTTGTATGGAG
UT6-e      645 GGTGGGTTGTTGTGGGTGGCGGGCTGCAGGGCTTGAAGAGTTGTATGGAG
consensus  651 ***********.*.***************.********.****.*****.

UT6-a      631 GGGCGTGTGATTGTATGTTGGTGTTGAGAGTGGGGATGGATGGGATGAGG
UT6-b      630 GGGCGTGTGATTGTATGTTGGTGTTGAGAGTGGGGGTGGATGGGATGAGG
UT6-c      670 GGGCGTGTGATTGTATGTTGGTGTTGAGAGTGGGGATGGATGGGATGAGG
UT6-d      694 GGGGGTGTGATTGTATGTTGGTGTTGGGAGTGGGGATGGATGGGATGAGG
UT6-e      695 GGGGGTGTGATTGTATGTTGGTGTTGGGAGTGGGGATGGATGGGATGAGG
consensus  701 ***.**********************.********.**************

UT6-a      681 GATTGAATGTGTCAACGGCTGGAGCGCTGGACGGGTCAACCGCTGATAGG
UT6-b      680 GATTGAATGTGTCAACGGCTGGAGCGCTGGACGGGTCAACCGCTGATAGG
UT6-c      720 GATTGAATGTGTCAACGGCTGGAGCGCTGGACGGGTCAACCGCTGATAGG
UT6-d      744 GATTGAATGTGTCAACGGCTGGAGCGCTGGACGGGTCAACCGCTGGACGG
UT6-e      745 GATTGAATGTGTCAACGGCTGGAGCGCTGGACGGG---------------
consensus  751 ***********************************...............

UT6-a      731 ----------------GTTAG------GGTTAGGG
UT6-b      730 ----------------GTTATGGTTAGGGTTAGGG
UT6-c      770 ----------------GTTAG------GGTTAGGG
UT6-d      794 CTCAACCGCTGATAGGGTTAG------GGTTAGGG
UT6-e      780 -TCAACCGCTGATAGGGTTAG------GGTTAGGG
consensus  801                 ****.      ********
```

TTAGGG

Supp. Fig. 2. *Sequences of UT6 STELA products*
The sequences of 5 clones derived from a UT6 STELA reaction are aligned. Highlighted in yellow are the UT6-F primer and the start of the TTAGGG repeats in each clone.

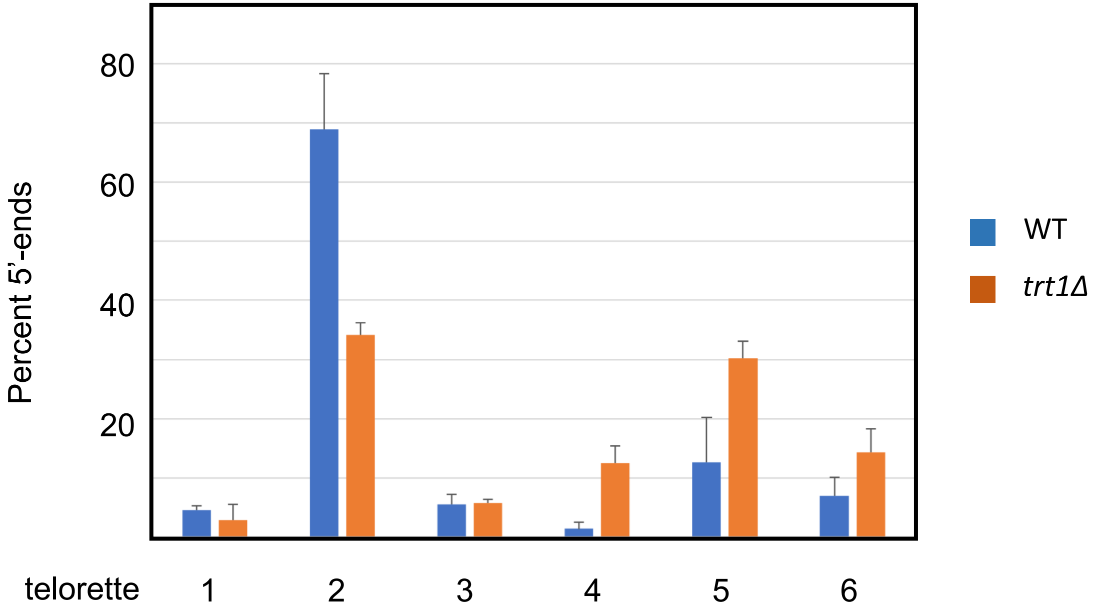

Supp. Fig. 3. *Telomere C-strand 5’-end distributions in wild type and trt1Δ strains*
The number of UT4/5 STELA products generate from wild type and *trt1Δ* DNA by each telorette oligo was determined, and plotted as the percentage of total STELA products. Data (average ± S.D.) are from three independent experiments.

References

1. Kojic M, Kostrub CF, Buchman AR, Holloman WK (**2002**). BRCA2 homolog required for proficiency in DNA repair, recombination, and genome stability in Ustilago maydis. **Mol Cell** 10(3): 683-691. doi: S1097276502006329 [pii].

2. Yu EY, Hsu M, Holloman WK, Lue NF (**2018**). Contributions of recombination and repair proteins to telomere maintenance in telomerase-positive and negative Ustilago maydis. **Molecular microbiology** 107(1): 81-93. doi: 10.1111/mmi.13866.

3. Mao N, Kojic M, Holloman WK (**2009**). Role of Blm and collaborating factors in recombination and survival following replication stress in Ustilago maydis. **DNA Repair (Amst)** 8(6): 752-759. doi: 10.1016/j.dnarep.2009.02.006.
